# Examining the Impact of Sonodynamic Therapy With Ultrasound Wave in the Presence of Curcumin-Coated Silver Nanoparticles on the Apoptosis of MCF7 Breast Cancer Cells

**DOI:** 10.1049/nbt2/7036856

**Published:** 2025-07-15

**Authors:** Zeinab Hormozi-Moghaddam, Ali Neshasteh-Riz, Seyedeh Mona Taheri, Seyed Mohammad Amini, Ehsan Sedghinezhad

**Affiliations:** ^1^Radiation Biology Research Center, Iran University of Medical Sciences (IUMS), Tehran 1449614535, Iran; ^2^Department of Radiation Sciences, Allied Medicine Faculty, Iran University of Medical Sciences, Tehran 1449614535, Iran; ^3^Microbial Biotechnology Research Center, Iran University of Medical Sciences (IUMS), Tehran 1449614535, Iran

**Keywords:** apoptosis, cancer, curcumin, nanoparticle, silver, ultrasound wave

## Abstract

**Introduction:** Sonodynamic therapy (SDT) is a promising approach that combines low-intensity ultrasound (LIUS) with a sensitizing agent to induce therapeutic effects. Curcumin-coated silver nanoparticles (Cur@AgNPs) have shown potential as a sensitizer, demonstrating adverse effects on cancer cell survival. This study examined the apoptotic effects of US waves in the presence of Cur@AgNPs on MCF7 breast cancer cells.

**Methods and Materials:** MCF7 cells were cultured and divided into different treatment groups. Cur@AgNPs were synthesized and characterized using various techniques, confirming their size to be approximately 29.3 ± 5.6 nm. The IC50 of Cur@AgNPs in MCF7 cells was determined to be 48.23 µg/ml through the MTT (3-(4,5-dimethylthiazol-2-yl)-2,5-diphenyltetrazolium bromide) assay. LIUS radiation was applied to the cells in different modes, both with and without Cur@AgNPs. Cell viability was evaluated using the MTT assay and reactive oxygen species (ROS) production was measured. Colony formation assay and real-time PCR were conducted to evaluate cell death and changes in gene expression of Bcl-2-associated X protein (Bax), B-cell lymphoma-2 (Bcl-2), and Caspase-3, respectively.

**Results:** The findings confirmed the successful synthesis of Cur@AgNPs with a uniform size of approximately 29.3 ± 5.6 nm. In the continuous wave (CW) and pulse wave (PW) modes, 50% and 25%, cell viability was measured at 65.01% ± 1.35%, 73.75% ± 1.80%, and 80.76% ± 1.57%, respectively. Cell viability in CW with Cur@AgNPs was 16.9% ± 4%. The plating efficiency (PE) of the combined treatment group was 13.66 ± 1.24, compared to 39.33 ± 1.24 for the US.CW group and 68.66 ± 2.62 for the Cur@AgNPs group. Also, the expression of proapoptotic genes, such as Bax and Caspase-3, increased, while the expression of the antiapoptotic gene Bcl-2 decreased in MCF7 cells treated with the SDT. Flow cytometry analysis revealed increased rates of early apoptosis (21.22% ± 3.82%) and late apoptosis (36.59% ± 4.5%) in the US.CW + Cur@AgNPs.

**Conclusion:** This study provides novel insights into the induction of apoptosis in MCF7 breast cancer cells through SDT in the presence of Cur@AgNPs as a sonosensitizer. These findings support the potential of SDT as an effective therapeutic approach for breast cancer treatment using nonionizing and noninvasive methods.

## 1. Introduction

Currently, breast cancer treatment primarily involves surgery, radiotherapy, and chemotherapy [1–3]. However, these methods are associated with toxicity, significant side effects, drug and radiation resistance, and high recurrence rates. Today, there is a growing emphasis on noninvasive and nonionizing methods that offer high therapeutic efficacy with minimal side effects. Among these emerging strategies are photodynamic therapy (PDT), targeted drug delivery systems, and nanoparticles synthesized through green chemistry approaches, all of which have shown great promise in preclinical and clinical settings. In parallel, ultrasound (US)-based therapies are gaining increasing attention due to their nonionizing nature, deep tissue penetration, and potential for precise tumor targeting. These advancements collectively mark a paradigm shift in the landscape of cancer treatment, focusing on safer, more effective, and patient-friendly approaches [4, 5]. US waves generate sonochemical reactions and produce free radicals in sonodynamic therapy (SDT) within cancer cells through the inertial interaction of cavitation in the environment. In recent years, nanoparticles have emerged as effective acoustic sensitizers due to their stability, biosafety, and variable size [6, 7]. Some research has demonstrated their potential to suppress tumors in organs like the pancreas, colon, lung, and brain [8–10]. Nanoparticles act as sensitizers, augmenting the production of free radicals and enhancing the efficacy of SDT in cancer cells. Several silver, copper, and gold nanoparticles have been shown to induce oxidative stress and apoptosis in mammalian cells [11, 12]. However, their lack of specificity and potential toxicity in healthy tissues have led researchers to explore the combination of metal nanoparticles with biological coatings and targeted methods, such as US waves, to overcome these limitations [13].

In addition, biological substances, including vegetable extracts and oils, have been employed to cover metal nanoparticles such as silver and gold [14, 15]. Curcumin, an active compound found in turmeric extracted from the *Curcuma longa* plant, is one such example [16, 17]. Curcumin is a natural flavonoid with broad antiviral, anti-infectious, and anticancer properties [18–20]. Its effect on apoptosis has garnered significant attention from researchers [21–23]. Therefore, curcumin's capability to influence apoptosis signaling pathways increases the sensitivity of cancer cells to treatment [24]. Biologically, curcumin increases redox reactions and the production of reactive oxygen species (ROS), leading to the upregulation of apoptosis receptors in tumor cell membranes. It also enhances the expression and activity of p53, which inhibits tumor cell proliferation and induces apoptosis [25–27]. Moreover, curcumin effectively inhibits the activity of NF-κB and COX-2, which are involved in the overexpression of anti-apoptotic genes like B-cell lymphoma-2 (Bcl-2) [28–31]. Previous studies have demonstrated the impact of silver nanoparticles (AgNPs) on increasing ROS production, halting the cell cycle and inducing apoptosis by downregulating Bcl-2, releasing cytochrome c, and activating caspase 9, 3, and PARP cleavage [32–35]. Combining curcumin with nanoparticles further enhances its therapeutic efficacy [36].

Specifically, AgNPs coated with curcumin (Cur@AgNPs) have attracted considerable interest due to their distinct characteristics and wide-ranging applications across various fields. Cur@AgNPs have exhibited significant anticancer effects in lung cancer and colon adenocarcinoma by inhibiting cell proliferation. Cur@AgNPs have been reported to reduce toxicity, increase stability, and induce apoptosis in cancer cells [37]. SDT has also been shown to reduce Bcl-2 expression, increase Bcl-2-associated X protein (Bax) expression, and induce apoptosis in cancer cells [38]. These genes (Bax, Bcl-2, and Caspase-3) were selected for analysis due to their central roles in the intrinsic (mitochondrial) apoptotic pathway, which is a key mechanism of cell death induced by ROS generated through presence of nanoparticle and US stimulation. Bax promotes mitochondrial outer membrane permeabilization (MOMP), facilitating the release of cytochrome c and the subsequent activation of downstream apoptotic signals. In contrast, Bcl-2 acts as an antiapoptotic regulator by preserving mitochondrial integrity and inhibiting cytochrome c release. The Bax/Bcl-2 ratio is widely recognized as a critical determinant of cellular commitment to apoptosis. Caspase-3, an essential executioner caspase, mediates the proteolytic cleavage of various cellular substrates, thereby executing the final stages of apoptosis. The expression profiles of these genes, thus, serve as reliable biomarkers for evaluating apoptosis induction in response to combined nanoparticle and US treatments [39, 40]. Previous studies have explored the use of SDT; however, its clinical application remains limited due to several challenges, including variability in treatment parameters and insufficient control over therapeutic outcomes. To address these limitations, precise optimization of the physical parameters of low-intensity US (LIUS) can enhance therapeutic efficacy by promoting sonosensitizer activation, increasing free radical generation and improving outcomes in sonochemical therapy. SDT primarily exerts its effects through acoustic cavitation, which significantly elevates intracellular ROS levels. This oxidative stress induces lipid peroxidation, DNA damage, and mitochondrial dysfunction, ultimately leading to apoptotic or necrotic cell death [41, 42]. So, this study aims to investigate the combined impact of SDT with LIUS in the presence of Cur@AgNPs on the expression of apoptosis-related assessments in breast cancer cells. By exploring the potential of US-mediated therapy using Cur@AgNPs, this research aims to contribute to developing novel strategies for breast cancer treatment which can surpass the limitations of current methods and enhance therapeutic outcomes.

## 2. Materials and Method

### 2.1. Cell Culture

The MCF-7 human breast cancer cell line was cultured in DMEM/F12 medium (Biowest Co., Nuaillé, France) with 10% fetal bovine serum at 37°C and a 95% humidity in 5% CO_2_ incubator (Model INCO108, Memmert co., Germany). The cultured cells were divided into 13 groups for study such as control (Ctrl), Sham (the MCF7 cell group was without applying the treatment conditions and without the treatment protocol), MCF7 cell groups were subjected to US with continuous mode (US.CW), and pulsed mode with duty factor (DF) 50%, 25%, 12%, and 6.25% in US. PW 50%, US. PW 25%, US. PW 12%, and US. PW 6.25%, respectively. In combination groups, MCF7 cell groups were exposed to the continuous and pulsed mode in the presence of Cur@AgNPs in groups US.CW + Cur@AgNPs, US. PW 50% + Cur@AgNPs, US. PW 25% + Cur@AgNPs, US. PW 12% + Cur@AgNPs, and US. PW 6.25% + Cur@AgNPs, respectively.

### 2.2. Preparation of Cur@AgNPs

To prepare Cur@AgNPs, we followed the protocol presented in our previous report [43]. Briefly, we added 200 µL of curcumin (C_21_H_20_O_6_, 65%, Sigma–Aldrich, USA, 40 mM in DMSO solvent) to 15 ml of deionized water (DIW, resistivity of 18.3 MΩ cm). After setting the pH in the range of 10, the solution is stirred for 3–5 min, and then, we add 2.5 ml of silver nitrate (AgNO_3_ 2.5 mM) to the curcumin solution. This solution is vigorously stirred with a magnet for 3 h and then, kept still at room temperature for 3 days. In order to remove the unreacted materials and the product, several washing procedures were performed according to the protocol provided for the preparation of curcumin-coated gold nanoparticles [44]. Characterization of the Cur@AgNPs was carried out using various techniques like transmission electron microscopy (TEM; Zeiss EM 900, Germany), UV-visible spectroscopy (model: NDNM96, NanoMabna Co, Iran), and dynamic light scattering (DLS; NANO-flex Particle Sizer, Germany). Inductively coupled plasma-optical emission spectroscopy (ICP-OES; the VISTA-PRO model from Varian Co., Australia) was employed to determine the concentration of the synthesized nanoparticles. The IC_50_ of Cur@AgNPs was evaluated after 24 h using the MTT (3- (4,5-dimethylthiazol-2-yl)-2,5-diphenyltetrazolium bromide) assay.

### 2.3. US Wave

In this study, MCF7 cells were first treated with Cur@AgNPs and incubated for 24 h to allow nanoparticle uptake and cellular interaction. Subsequently, SDT using US waves was performed to investigate the optimal treatment protocol. A circular flat transducer (Phyaction 190i, Germany) operating at a frequency of 1 MHz was employed for US irradiation. Both continuous wave (CW) and pulsed wave (PW) radiation were applied, with varying DFs of 50%, 25%, 12%, and 6.25%. The acoustic intensity of 2 W/cm^2^ was employed to eliminate the nonlinear effect. The exposure time was determined by monitoring the temperature increase by one degree during US treatment. Employing these experimental parameters aimed to optimize the SDT for treating MCF7 cells in combination with Cur@AgNPs.

### 2.4. Cell Viability Assay

The survival of MCF7 cells was employed by MTT assay. To prepare the MTT solution, 5 mg of its powder was dissolved in 1 ml of phosphate-buffered saline (PBS) and filtered through a 0.20 μm filter. The solution was stored in the dark at −4°C for 6 months. To evaluate cell viability in noncombined and combined groups (with specified parameters) with IC_50_ of Cur@AgNPs and US, 50,000 MCF7 cells were seeded in 24-well plates. The cells were incubated for 4 h with the MTT solution. Subsequently, a cell viability assay was conducted using a DANA ELISA reader (Model-DA3200, DANA Co., Iran) at a wavelength of 570 nm. The cell viability results were reported as a relative decrease in absorbance compared to the control (Ctrl) group. Images of the nuclei were captured and analyzed for any changes or effects resulting from the experimental treatments by DAPI (VECTASHIELD, Antifade Mounting Medium with DAPI (H-1200-10), U.S.A) staining. Fixed cell culture samples were prepared. After staining and drying, the cell plates were examined using a fluorescence microscope (Nikon Instrumemt Co, U.S.A).

### 2.5. ROS Assay

The subsequent procedure was utilized to measure the levels of free radical generation induced by the SDT technique. MCF7 cells were incubated with 100 μM of 2′,7′-dichlorofluorescein diacetate (DCFH-DA; Pazohan Razi (TPR) Co., Iran) for 30 min. Subsequently, the DCFH-DA solution was removed by washing the cells with PBS. A fluorescent spectrophotometer equipped with a screen reader capability (the Perkin Elmer Fluorescent Spectrophotometer) was utilized to an excitation wavelength of 485 nm and an emission wavelength of 530 nm. The obtained data were compared to a positive control group (Ctrl+; MCF-7 cells treated with 100 nM hydrogen peroxide (H_2_O_2_)) and a negative control group (Ctrl−; MCF-7 cells without treatment and H_2_O_2_). This comparison allowed for assessing the generation of free radicals prompted by the SDT technique by the US treatment method in the presence of Cur@AgNPs.

### 2.6. Colony Formation Assay

A total of 100 cells were cultured in 5 ml DMEM/F12 medium and appropriate incubation conditions for 14 days to assess the colony formation rate in different treatment groups. Then, colonies were fixed using paraformaldehyde and dyed with crystal violet. Colonies were counted using an inverted microscope (Nikon) and Image J software (the National Institutes of Health in Bethesda, Maryland, USA), and plating efficiency (PE) was analyzed.

### 2.7. Real-Time PCR

The noncombined and combined cell groups are extracted after different treatments to examine alteration in the expression of Bax, Bcl-2, and Caspase-3 genes. Trizol will extract whole-cell RNA in accordance with the instructions provided by the manufacturer. RNA will be checked by examining the sample on a 2% agarose gel and a Nanodrop device (nanomabna Co., Tehran, Iran). To evaluate the real time-PCR method, specific primers for cDNA sequence synthesis will be assessed based on previous studies and recorded in the NCBI database (Table 1). A total of 1000 ng/ml RNA of each sample will be used for cDNA synthesis according to the instructions of SYBR Premix Ex Taq (Perfect RealTime) made by Takar Co., Japan. Glyceraldehyde-3-phosphate dehydrogenase (GAPDH) will be used as a reference gene to compare and check the correct expression of the target gene.

### 2.8. Flow Cytometry

The propidium iodide (PI)/annexin V-FITC Apoptosis-Necrosis detection kit (BioLegend Co., Cat number: 640930, USA) was employed to assess the proportion of apoptotic and necrotic cells within the cell population, following the guidelines provided by the manufacturer. The treatment groups, both combined and noncombined, were examined and juxtaposed with the control using flow cytometry (Becton Dickinson BD, FACS Calibur, U.S.A).

### 2.9. Statistical Analysis

The results obtained from the cellular phase, including cell, ROS assay, colony formation assay, gene expression, and apoptosis, are presented as mean ± standard deviation. At least 50,000 MCF7 cells will be cultured in each cell group. Different treatment protocols will be compared across cell groups, with three samples from each group as a guide. Statistical analysis will be conducted utilizing SPSS software, employing one-way analysis of variance (ANOVA), with a confidence level of 95% and a significance threshold set at *p*  < 0.05.

## 3. Results

### 3.1. Characteristic of Cur@AgNPs

**Figure 1:**
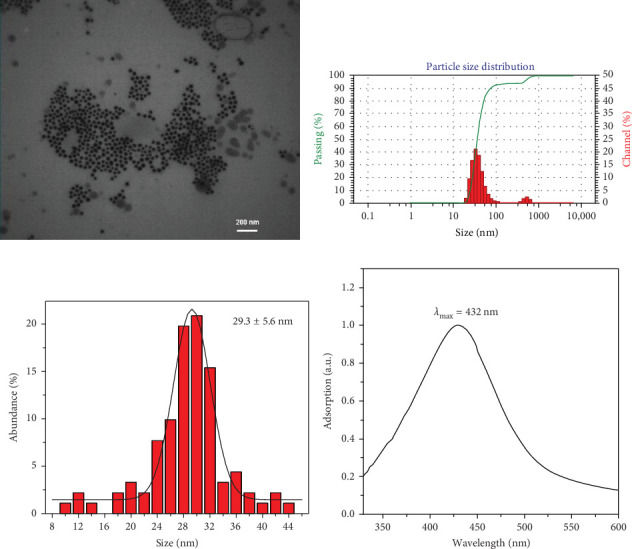
Characteristics of Cur@AgNPs: (A) transmission electron microscope (TEM) image, (B) dynamic light scattering (DLS) analysis, (C) quantification analysis of diameter, and (D) UV-visible spectrum.

The study's results demonstrated the significant effect of SDT on MCF-7 cell death and induction of apoptosis in the presence of Cur@AgNPs. Nanoparticles were characterized and confirmed by various techniques (Figure 1A–D).

Figure 1A indicates that TEM analysis was performed to determine the size and morphology of the synthesized Cur@AgNPs. The TEM image revealed that the nanoparticles exhibited a uniform spherical shape. Figure 1B shows DLS analysis to determine the hydrodynamic size and size distribution of the Cur@AgNPs in a liquid suspension. The diameter of the nanoparticles was approximately 29.3 ± 5.6 nm, consistent with the TEM results (Figure 1B,C). The UV-visible absorption spectrum exhibited a characteristic peak at around 432 nm (Figure 1D). Also, the zeta potential (Zeta-check, Microtrac, Germany) measurement showed a negative surface charge, indicating stabilizing agents on the nanoparticle surface.

### 3.2. Cell Viability

**Figure 2:**
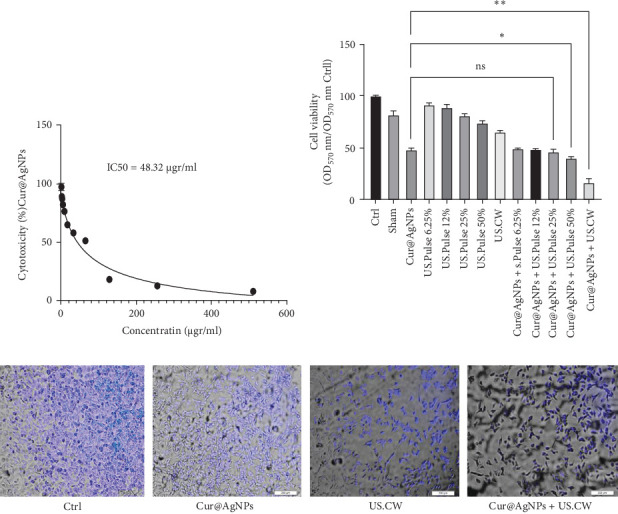
The impact of SDT on MCF7 cells when combined with Cur@AgNPs in both pulsed and continuous modes. (A) Determining the IC50 of Cur@AgNPs nanoparticles at varying concentrations (μg/ml), (B) assessing cell viability following the treatment, and (C) conducting DAPI staining in different experimental groups, including control (Ctrl), Cur@AgNPs, US.CW, and Cur@AgNPs + US.CW (merge with Photoshop Software).

The cells were treated with sensitizers and incubated for 24 h to evaluate the cytotoxic effect of Cur@AgNPs in MCF7 cells. IC_50_ concentration of Cur@AgNPs was used for this study (Figure 2A). SDT was performed using an US frequency of 1 MHz, intensity of 2 W/cm^2^, and a distance of 2 cm. The irradiation time was varied in continuous mode (CW) and in pulse mode (PW) for 50%, 25%, 12%, and 6.25% duty cycles with for 35 ± 0.05, 50 ± 0.02, 60 ± 0.05, 80 ± 0.03, and 150 ± 0.02 s, respectively. The effect of SDT in the presence of Cur@AgNPs decreases cell viability dependence on US mode (Figure 2B,C).

The MTT assay indicated that the combination of US and Cur@AgNPs significantly reduced cell viability compared to Ctrl and US-only groups in IC50 48.32 µg/ml. In the continuous mode (CW) treatment, the cell viability was measured at 65.01% ± 1.35%, and with Cur@AgNPs 16.9% ± 4%. In the pulse mode (PW) treatment, cell viability was assessed under different duty cycle percentages, namely, 50% and 25% with 73.75% ± 1.80%, and 80.76% ± 1.57%. The results demonstrated that increasing the duty cycle percentage with the presence of Cur@AgNPs reduced cell viability. After treatment with 50% and 20% duty cycle US, the cell viability decreased to 39.9% ± 1.34% and 45.97% ± 2.52% (Figure 2B). Additionally, DAPI staining was conducted to evaluate the cellular morphology and nuclear changes in the different experimental conditions (Figure 2C). The control group represented the untreated cells, while Cur@AgNPs, US.CW, and Cur@AgNPs+US.CW groups received specific treatments. The staining patterns were examined and compared to identify differences in nuclear characteristics and overall cellular appearance.

### 3.3. ROS Assay

**Figure 3:**
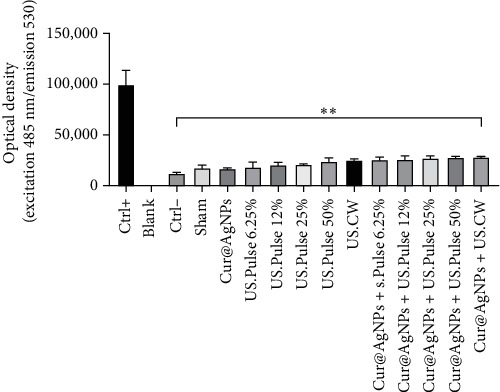
The ROS free radicals generated by SDT in the presence of Cur@AgNPs was assessed using the fluorescence signal of DCFH-DA solutions, measured based on optical density (OD) (*⁣*^*∗∗*^*p* < 0.01).

Considering the potential role of ROS in oxidative stress, a fluorescent probe with optical density (OD) was used to investigate its levels in breast cancer cells. Combined treatment increased the amount of ROS induction (Figure 3).

Figure 3 indicates a significant increase in the SDT groups compared to the Ctrl group. This increase shows a higher production of ROS in the Cur@AgNPs + US.CW with 2.3 times Ctrl− (negative) suggests an induction of oxidative stress.

### 3.4. Colony Formation Assay

**Figure 4:**
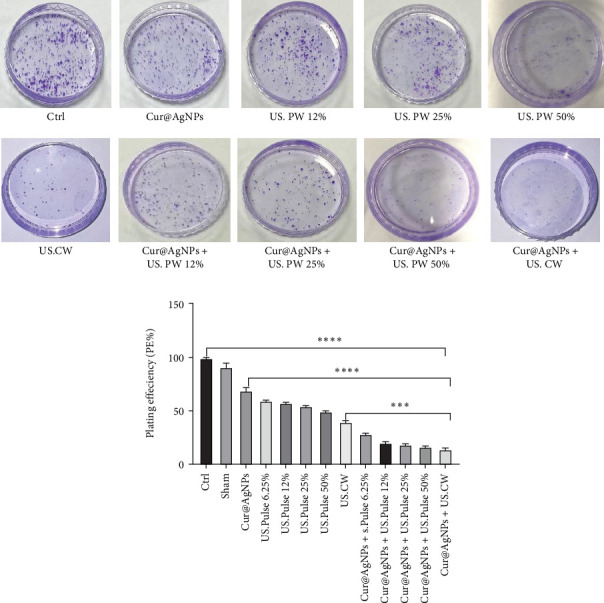
Colony formation assay. (A) Morphological characteristics of colonies in MCF7 cell groups and (B) plating efficiency (PE) of MCF7 cells (*⁣*^*∗∗∗*^*p* < 0.001 and *⁣*^*∗∗∗∗*^*p* < 0.0001).

Assessment of survival in MCF7 cells subjected to CW US and Cur@AgNPs exhibited a notable reduction in colony formation capacity compared to both the Ctrl and noncombined treatment groups.  Figure 4A,B depicts the PE (PE%) results obtained from the combined treatment.

The rate of cell death and colony formation of PE in the Ctrl and Sham groups were 99 ± 0.81 and 90.66 ± 3.29. A significant decrease is indicated in the US.CW + Cur@AgNPs combined treatment group with 13.66 ± 1.24 compared with the US.CW and Cur@AgNPs were, respectively, 39.33 ± 1.24 and 68.66 ± 2.62. This increase can be attributed to cell death by combination therapy.

### 3.5. Real-Time PCR

**Figure 5:**
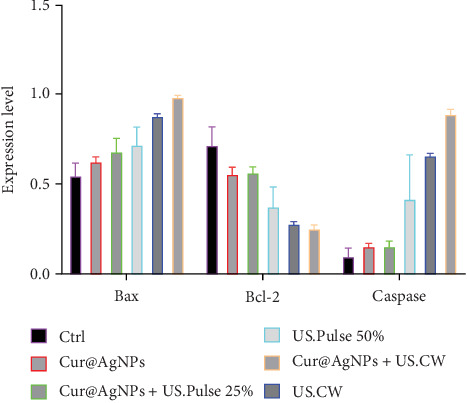
The levels of expression for the Bax, Bcl-2, and Caspase3 genes under various treatments are depicted in the legend.

The results obtained from real-time to investigate the expression levels of Bax, Bcl-2, and Caspase-3 genes under the influence of LIUS treatment and Cur@AgNPs, indicate an increase in the expression of proapoptotic genes, such as Bax and Caspase-3 and a decrease in Bacl-2 as an antiapoptotic gene expression (Figure 5).

The findings show that the expression of Bax, Bcl-2, and Caspase-3 genes in MCF7 cells of the Ctrl group, respectively, and in the presence of LIUS continuously were 0.55 ± 0.05 and 0.72 ± 0.08, respectively. In addition to the data analysis, showed the increase in Bax and Caspase-3 expression with 0.98 ± 0.05 and 0.89 ± 0.01 and the decrease in Bcl-2 expression 0.25 ± 0.01 after SDT in CW mode within the presence Cur@AgNPs.

### 3.6. Flow Cytometry Analysis

**Figure 6:**
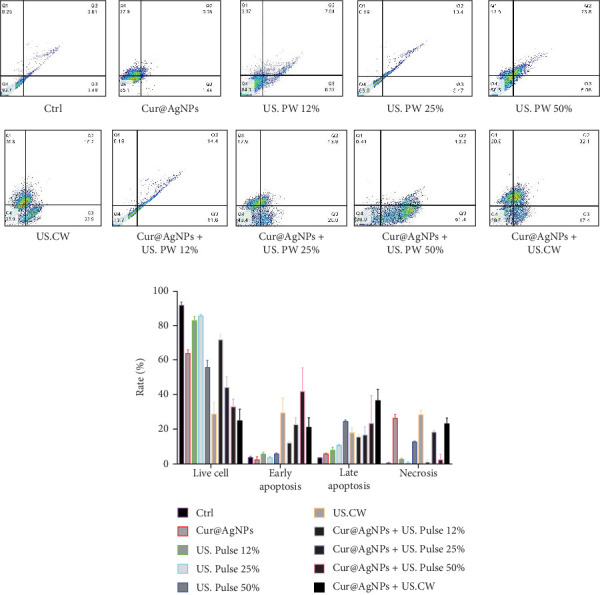
Flow cytometry was employed to analyze apoptosis and necrosis in MCF7 cells across treatment groups: (A) display of flow cytometry data and (B) quantitative assessment of live cells, early apoptosis, late apoptosis, and necrotic cell populations.

Flow cytometry with annexin and PI staining showed an increase in early and late apoptosis rates in the combined treatment of Cur@AgNPs and LIUS radiation (Figure 6A). The results of this investigation on MCF7 cells treated with CW and Cu@NPs showed 21.22% ± 3.82% and 36.59% ± 4.5% for early and late apoptosis, respectively, and 23.2% ± 2.3% for necrosis (Figure 6B).

The evaluation results of early and late apoptosis and necrosis in MCF7 cells of the Ctrl group were 3.77 ± 0.32, 3.55 ± 0.05, and 0.50 ± 0.25, respectively. In the presence of Cur@AgNPs, early and late apoptosis were 2.53 ± 1.09 and 5.79 ± 0.23 (Figure 6B).

The rates of early and late apoptosis and necrosis in the US. PW 50% US were 5.55 ± 0.49, 24.41 ± 0.61, and 12.8 ± 0.20, respectively. However, with the combination of the US. PW 50% and Cur@AgNPs, these rates significantly increased to 41.95 ± 9.45, 23.45 ± 11.25, and 2.48 ± 2.07, respectively. This significant increase in early apoptosis in the CW treatment group, among other results, demonstrates the efficacy of the combined treatment and holds promise for potential therapeutic approaches in breast cancer treatment, highlighting the potential of our research.

## 4. Discussion

LIUS combined with sensitizing molecules known as SDT has been used in various therapeutic fields. SDT has been recognized as a promising noninvasive and nonionizing approach that produces antitumor effects for cancer treatment. SDT uses US-induced cavitation and sonosensitizers to create free radicals that rapidly affect cancer cells [45]. As the US waves pass through the tissue, they interact with the matter and cause changes that result in mechanical and thermal interactions. The most biological impact is caused by mechanical effects such as radiation force, microstreaming, and acoustic cavitation. Acoustic cavitation is an important effect of mechanical interaction which occurs at low frequencies and intensities, leading to the production of free radicals and increasing the effectiveness of SDT [46, 47]. Also, one of the key advantages of SDT is its ability to treat deep tumors, as it uses low-frequency US [45, 48]. And an optimal combination of low-intensity, low-frequency US and a sensitizing agent forms the basis of SDT for the transition of sonosensitizers from a nontoxic to a toxic state only in the presence of ROS [49].

Nanoparticles and biosynthesized materials have attracted increasing attention in different approaches to breast cancer treatment [50, 51]. For example, Das et al. [52] studied the successful treatment of MCF-7 cells using gold nanoparticles biosynthesized. In another study, Karthick et al. [53] reported the induction of apoptosis using PLGA microspheres loaded with quercetin as biodegradable microspheres. Also, AgNPs have been extensively studied for their adverse effects on treatment.

The potential benefits of combining SDT with other therapeutic modalities such as photothermal therapy (PTT) and chemotherapy have been increasingly recognized. These combinational strategies aim to achieve a synergistic enhancement of therapeutic efficacy while minimizing adverse effects. An essential factor in maximizing treatment outcomes is the selection of biocompatible, green-synthesized nanoparticles with high bioavailability, which not only enhance cancer treatment but may also offer antimicrobial properties. In one study, ZnO@Ag nanorods demonstrated significantly enhanced ROS production under US exposure, attributed to improved electron–hole separation efficiency. This ROS overproduction led to marked inhibition of tumor growth in vivo, highlighting the potential of such nanostructures as effective agents in SDT–based cancer therapy [54]. Curcumin nanoparticles exhibit various antioxidant, anti-inflammatory, antifungal, and antibacterial effects [55, 56]. Additionally, curcumin inhibits tumor growth, progression, and metastasis [57].

Tumors exhibit unique characteristics in their vascular system, capillary permeability, and lymphatic drainage, collectively referred to as the enhanced permeability and retention (EPR) effect [58, 59]. This property can be exploited to deliver nanoparticles ranging from 20 to 200 nm to the tumor site [60]. In this study, the TEM analysis confirmed the successful synthesis of well-dispersed Cur@AgNPs with an average size of 29.3 ± 5.6 nm. The UV-visible analysis confirmed the successful formation of Cur@AgNPs and their optical properties (Figure 1). However, curcumin's poor solubility in water and low bioavailability hinder its clinical utility [61]. Recent advancements in nanoparticle and US-based delivery systems have improved the bioavailability and solubility of curcumin [62, 63].

The present study aimed to evaluate the impact of different modes of LIUS on the MCF7 cancer cells in the presence of Cur@AgNPs. Our observations revealed a significant reduction in cell survival in the group treated with CW mode with IC_50_ of Cur@AgNPs (Figure 2A). These results indicate that SDT using a frequency of 1 MHz, intensity of 2 W/cm^2^ and various exposure times in both CW and PW modes can effectively decrease cell viability. These findings highlight the potential of SDT, particularly in combination with Cur@AgNPs, as a promising approach to cancer treatment. Also, in the control group, DAPI staining revealed a consistent and regular distribution of cell nuclei. However, in the treatment group exposed to the US.CW, Cur@AgNPs, and US.CW + Cur@AgNPs, a noticeable decrease in nuclei was observed, suggesting a potential impact on cell viability by the combined treatment (Figure 2B). In a similar study, curcumin-loaded silver nanoparticles synthesized via green methods were tested against MM-138, FM-55, and MCF-7 cell lines. For MCF-7, the IC_50_ values were 144.6 µg/mL for AgNPs, 81.2 µg/mL for curcumin, and 60.6 µg/mL for curcumin-loaded AgNPs, showing enhanced anticancer potential [64]. In the study by Tao et al. [54] using ZnO@Ag nanoparticles, it was reported that at a concentration of 50 µg/mL, approximately 30% of cancer cells were eliminated. From a biological standpoint, the most effective concentration was determined to be 25 µg/mL. Moreover, under US exposure as part of SDT, the cell viability was observed to be around 50%, indicating moderate therapeutic efficacy. In comparison, the use of Cur@AgNPs in our study at a concentration of 48.32 µg/mL resulted in an IC_50_ and a significantly lower cell viability of 16.9% under US. These findings suggest that Cur@AgNPs exhibit superior efficiency in inducing cancer cell death through SDT compared to Zn-based nanomaterials [54]. Motafeghi et al. [65] revealed that a silver-graphene nanocomposite inhibited MCF-7 cancer cell growth by 84.60% and increased ROS and lipid peroxidation levels by up to 74% and 70%, respectively. Wang et al. [66] investigated the effect of US in the presence of AgNPs on MCF-7 breast cancer cells. Their results demonstrated increased ROS production and cell death effects. Higher concentrations of AgNPs (100 μg/ml) acted as acoustic sensitizers and enhanced ROS production [66]. Effective results with curcumin can be achieved at lower concentrations when it is delivered using the appropriate nanoparticle formulation. Similarly, the combined treatment group (CW US and Cur@AgNPs) exhibited a significant increase in fluorescence intensity compared to the control group (Figure 3). Using curcumin as a biological coating reduced the toxicity of AgNPs and improved their significant effects with SDT [66].

The colony assay was continuously performed to evaluate the PE, representing the ability of individual cells to form colonies. The cells from the Ctrl and Sham groups exhibited a high PE of 99% (Figure 4A). In contrast, a significant decrease in PE was observed in the treatment group exposed to CW mode and PW 50% with the presence of Cur@AgNPs. The number of colonies formed was noticeably reduced, indicating an impairment in the ability of cells to proliferate and form colonies under the influence of the combined treatment (Figure 4B). Mohd Bohari et al. [67] evaluated the effects of LIUS on MCF-7 breast cancer cells using a frequency of 1 MHz and an intensity of 0.1 W/cm^2^ for 10 min per day over 3 days. The results showed that LIUS significantly reduced cell proliferation, increased apoptosis, and increased p53-mRNA expression, indicating selective induction of apoptosis in cancer cells with minimal damage to control cells [67]. Therefore, in this study, US waves with a frequency of 1 MHz and a low intensity of 0.50 W/cm^2^ were applied for 120 s, aiming to induce acoustic cavitation while maintaining thermal control.

Apoptosis induction using LIUS as a therapeutic approach has been extensively studied. For instance, Hassan et al. [68] analyzed the effects of Sanazole as a sound sensitizer based on cell type and sound parameters. US waves with a frequency of 1 MHz, applied in CW and PW modes at low intensities for 30 min, decreased cell survival, increased apoptosis, and elevated production of free radicals [68]. Chen and Zhang [38] explored the effect of US waves combined with drug-loaded microbubbles on tumor cell death, demonstrating that this approach promotes apoptosis in cancer cells by regulating the expression of Bcl-2 and Bax. The study demonstrated an upregulation of Bax and a downregulation of Bcl-2, indicating the activation of the mitochondrial (intrinsic) apoptotic pathway [38]. Notably, this proapoptotic response is closely linked to elevated levels of ROS, which are well-known for inducing oxidative stress and compromising mitochondrial membrane integrity. The resulting damage leads to a reduction in mitochondrial membrane potential (MMP) and the opening of mitochondrial permeability transition pores (mPTPs), ultimately facilitating the release of cytochrome c from the mitochondria into the cytosol. Once released, cytochrome c interacts with apoptotic protease activating factor-1 (Apaf-1) to form the apoptosome complex, which subsequently activates caspase-9, followed by the activation of Caspase-3, executing the final steps of apoptosis [38, 42].

Similarly, in the present study, the Bax and Caspase-3 expression in the combined treatment group were significantly higher than in the Ctrl group, indicating enhanced apoptosis induction (Figure 5). In the context of the study, flow cytometry analysis of MCF7 cells treated with the combined method showed a significant increase in early and late apoptosis rates (Figure 6A,B). In the study by Karthick et al. [53], using PLGA, the late apoptotic rate at the highest concentration was estimated to be 6.78%. In comparison, the late apoptotic rate in the Cur@AgNPs group was 5.79%, while the US treatment group showed a significantly higher rate of 36.59%.

The results demonstrated that the combined treatment significantly reduced cell survival and increased apoptosis rates compared to the control group. The Bax/Bcl-2 ratio, indicative of apoptosis induction, was also higher in the combined treatment group with the US.CW and US. PW with a high-DF. In a similar study conducted by this team, the use of US waves with green nanoparticles can have a significant impact on the cancer treatment process [69].

These findings suggest that using LIUS in combination with Cur@AgNPs as an SDT method could be a promising approach for enhancing the therapeutic efficacy of curcumin in breast cancer treatment. Additionally, the safety and potential side effects of using Cur@AgNPs should be thoroughly evaluated before considering its clinical application.

## 5. Conclusion

In this study, the parameter conditions for US were carefully determined. Therapeutic US as an SDT method was applied at a frequency of 1 MHz. These results suggest that combining US with CW and PW 50% in the presence of Cur@AgNPs can decrease cell survival, inhibit colony formation, increase ROS production, and induce apoptosis in the targeted cells. However, more research is needed to fully comprehend the underlying mechanisms and assess the safety and effectiveness of this combined treatment strategy.

## Figures and Tables

**Table 1:** The primer sequences used in real-time RT-PCR.

Primer name		Sequence (5′ to 3′)
Bax	Bax-F	GGAGCTGCAGAGGATGATTGCC
	Bax-R	TCCCGCCACAAAGATGGTCACG

Bcl-2	Bcl2-F	GATACTGAGTAAATCCATGCAC
	Bcl2-R	AGTGTTGCAGAATATCAGCCAC

Caspase-3 (CASP3)	Casp3-F	GGGCCTACAGCCCATTTCTCC
	Casp3-R	GCCGTCTAGAGTCCTATGTGC

## Data Availability

No new data were created or analyzed during this study. Data sharing is not applicable to this article.
